# Federated knee injury diagnosis using few shot learning

**DOI:** 10.3389/frai.2025.1589358

**Published:** 2025-07-23

**Authors:** Chirag Goel, Anita X, Jani Anbarasi L

**Affiliations:** School of Computer Science and Engineering, Vellore Institute of Technology, Chennai, India

**Keywords:** federated learning, few shot learning, knee MRI, medical image, MRNet

## Abstract

**Introduction:**

Knee injuries, especially Anterior Cruciate Ligament (ACL) tears and meniscus tears, are becoming increasingly common and can severely restrict mobility and quality of life. Early diagnosis is essential for effective treatment and for preventing long-term complications such as knee osteoarthritis. While deep learning approaches have shown promise in identifying knee injuries from MRI scans, they often require large amounts of labeled data, which can be both scarce and privacy-sensitive.

**Methods:**

This paper analyses a hybrid methodology that integrates few-shot learning with federated learning for the diagnosis of knee injuries using MRI scans. The proposed model used a 3DResNet50 architecture as the backbone to enhance both feature extraction and embedding representation. A combined Centralized and Federated Few-Shot Learning Framework is analysed to leverage episodic-intermittent training strategy based on Prototypical Networks. The model is trained incorporating Stochastic Gradient Descent (SGD), Cross-Entropy Loss, and a MultiStep Learning Rate scheduler to enhance few-shot classification. This model also addressed the challenge of limited annotated data ensuring patient data privacy through distributed learning across multiple regions.

**Results:**

The models performance was evaluated on the MRNet dataset for multi-label classification. In the centralized setting, the model achieved accuracies of 85.3% on axial views, 82.1% on sagittal views, and 71% on coronal views. The propose work attained accuracies as 83% (axial), 83.9% (sagittal), and 65% (coronal), demonstrating the framework’s effectiveness across different learning configurations.

**Discussion:**

The proposed method outperforms in diagnostic accuracy, generalization across MRI planes, and patient privacy via federated learning. However, it faces limitations, including lower coronal view performance and high computational demands due to its complex architecture.

## 1 Introduction

The knee joint is essential for both mobility and stability but is particularly vulnerable to injuries like anterior cruciate ligament (ACL) tears and meniscal damage. ACL which is a major stabilizing ligament in the knee, is often torn during sudden turning or twisting gestures, leading to symptoms like pain, swelling, and joint instability. Similarly, the meniscus, a C-shaped cartilage that cushions the knee also suffers injury from abrupt movements or gradual wear, resulting in discomfort and limited range of motion. These types of injuries are frequently found in athletes and often necessitate clinical treatment, which may include physical rehabilitation, bracing, or surgery. While advancements in imaging techniques and medical interventions have enhanced diagnostic capabilities, attaining early and accurate identification of ACL and meniscus injuries remains a significant clinical obstacle. Misdiagnosis or delays in treatment can degrade outcomes, accelerating the development of knee osteoarthritis and recovery. Maniar et al. (2022) observed a growing incidence of knee injuries, with ACL tears and knee contusions being the most prevalent particularly among female athletes.

According to the study by 2030–2031, there will be a sharp increase in ACL injuries, which is predicted to double from 2017 to 2018 levels and reach a worrying rate of 77.2 per 100,000 people. The increase in ACL injuries will result a financial burden in addition to a health risk because the standard treatment involves surgery and long rehabilitation periods often last more than a year. In fact, it is predicted that ACL injuries will cost around $236 million between 2030 and 2031. Additionally, there is a concerning trend among young Australians, specifically females between the ages of 5 and 14 where a sharp rise in the annual incidence of ACL injuries have been observed.

Poulsen et al. (2019) highlighted the critical role of early diagnosis in knee injuries, observing a significantly higher risk of knee osteoarthritis (OA) following ACL damage. Their study found that individuals with an ACL tear are nearly four times more likely to develop OA, and the risk increases to six times when both the ACL and meniscus are affected. Convolutional Neural Networks (CNNs) have proven effective in analyzing MRI scans for detecting such injuries by accurately identifying key anatomical features. However, CNNs require large annotated datasets and often fails to identify rare or unfamiliar cases.

Few-shot learning effectively addresses the limitations of traditional CNNs in medical imaging by demanding only a small number of labeled data, making it ideal for identifying rare knee injuries. The strength lies in the capability to identify classes with minimal data, which is very helpful for medical analysis. Rieke et al. (2020) detailed the need for large, diverse datasets for reliable deep learning models in healthcare, but data collection is often restricted by privacy concerns. Federated learning provides a better solution by enabling collaborative training across institutions without sharing raw patient data. This decentralized method enhances model performance while ensuring data privacy and compliance with ethical and legal standards.

The major contributions of this work are as follows:

- A hybrid approach is proposed that integrates Few-Shot Learning with Federated Learning for knee injury diagnosis from MRI scans. This integration overcomes the challenges caused due to limited labeled data thus enhancing diagnostic capabilities across distributed data sources.
- The proposed federated learning framework guarantees patient data privacy by maintaining a decentralized structure, while enabling efficient training across multiple institutions.
- Validation on the MRNet dataset demonstrates that the proposed model surpasses existing state-of-the-art methods for knee injury diagnosis, highlighting its real-world applicability.
- The data structuring approach supports multi-label classification by covering diverse injury types, allowing the model to detect and predict multiple conditions simultaneously, thereby enhancing both diagnostic precision and flexibility.

The structure of this paper is organized as follows: Section 2 details the related work proposed in Knee OA. Section 3 describes the proposed architecture of the Federated Few-Shot Knee Injury Diagnosis system. Section 4 outlines the implementation and presents the results for detailing the efficiency of the proposed model. Section 5 concludes the summarizing the key findings.

## 2 Related works

Tsai et al. (2020), presented a convolutional neural network architecture called the Efficiently-Layered Network (ELNet) for knee MRI diagnostic for triage. They found that ELNet can identify tears even in the absence of explicit localization information and also uses a single image stack (axial or coronal) as input. ELNet achieves an ROC-AUC score of 0.904 for Meniscus Tear, 0.96 for ACL Tear, and 0.941 for Abnormality detection when evaluated on the MRNet dataset. Azcona et al. (2020) utilized ResNet versions (18, 50, or 152) in place of the AlexNet model and transfer learning using ImageNet’s pre-trained weights resulting in better performance. Notably, the final layer is altered to predict results rather than using categorical vectors. Slices are fed one at a time during training, and the ultimate result is the maximum probability across all slices. The paper achieves a 0.934 combined AUC score on the validation data of the MRNet dataset. Dai et al. (2021) proposed a novel TransMed framework that combines self-attention mechanisms and transformer-based architecture for multi-modal medical image classification. It consists of two branches: a CNN branch for feature extraction and a transformer branch for capturing long-range dependencies within sequences. MRNet dataset the TransMed model obtained an ROC-AUC score as 0.952 for Meniscus Tear, 0.981 for ACL Tear, and 0.976 for Abnormality detection, respectively.

Joshi and Suganthi (2022) proposed a parallel deep convolutional neural network (CPDCNN) to improve feature distinctiveness in knee MRI images for the purpose of ACL tear detection. The CPDCNN attained an ROC-AUC of 0.952 for Meniscus Tear, 0.981 for ACL Tear, and 0.976 for Abnormality detection along with 96.60% accuracy for ACL tear diagnosis on the MRNet dataset. Yan et al. (2023) performed adversarial data augmentation for improving knee MRI classification. Through adversarial data augmentation techniques, this work achieved a combined AUC score of 0.8953% on the MRNet dataset. Hanin Al Al Ghothani and Zhang (2023) proposed a compact model for multi-label classification unlike traditional methods that uses multiple models for binary classification. To improve classification accuracy, the study uses data fusion, feature fusion that makes use of Convolution Block Attention Module (CBAM) attention, and decision fusion attaining a combined AUC score of 0.925% on the MRNet dataset.

Kara and Hardalaç (2021) proposed a CNN enhanced with auto encoder models to recognize anomalies or diagnoses of diseases attaining an ROC-AUC score of 74.5 for Meniscus Tear, 0.86 for ACL Tear, and 0.86 for Abnormality detection on the MRNet dataset. Bien et al. (2018) developed a deep learning model called MRNet to automatically analyze knee MRI scans obtaining impressive AUC scores of 0.847 for Meniscus Tear, 0.965 for ACL, and 0.937 for Abnormality. Hung et al. (2023) proposed a deep learning model for the automatic detection of meniscus tears in knee magnetic resonance imaging (MRI) using an improved YOLOv4 model with a backbone as Darknet-53 architecture attaining an accuracy of 78.8% on the MRNet dataset.

Li et al. (2023) proposed a deep learning technique to identify tears in the Anterior Cruciate Ligament (ACL) in knee MRI scans. Using a 3D weighted multi-view convolutional neural network, the model analyzed T1-sagittal, T2-sagittal, T2-coronal, and T2- transverse views of the knee joint. The model achieved an AUC score for ACL tear detection as 92.86% for, MRNet dataset. Belton et al. (2021) proposed the validation of localization and improvement of knee injury identification through spatial attention mechanisms integrated with CNN as MPFuseNet network. MPFuseNet analysed different fusion strategies for each of the network’s planes to identify the best one. The MPFuseNet achieved an AUC score of 0.957 for Abnormality, 0.977 for ACL, and 0.831 for Meniscus Tear on the MRNet dataset. Javed Awan et al. (2021) proposed modified 14-layer CNN architecture implemented with random splitting and validated using 3 and 5-fold cross-validation techniques. CNN-ResNet-14 model improved image diversity by utilizing real-time data augmentation. Furthermore, in order to enhance the distribution of unbalanced classes and bolster the model’s dependability in ACL tear detection, hybrid class balancing technique was implemented. Real-time data augmentation and hybrid class balancing was incorporated together to prevent overfitting and preserved model efficiency attaining an accuracy of 92% on the Knee MRI dataset.

Li et al. (2022) analysed Mask R-CNN for detecting and classifying meniscus tears in knee MRI scans. This model accurately segmented meniscus and cartilage regions, achieving an accuracy of 87.5% for healthy, 86.96% for torn, and 84.78% for degenerated menisci using 924 MRI images collected from eight hospitals. Dunnhofer et al. (2022) introduced MRPyNet, a model integrating a Feature Pyramid Network into MRNet and ELNET architectures attaining 88.6% accuracy for ACL tear detection, and 88.1% with ELNET for MRNet dataset. For meniscus tear classification, the MRNet–MRPyNet and ELNET–MRPyNet combinations achieved 77.8 and 76.1% accuracy, respectively.

Chang et al. (2019) analysed complete anterior cruciate ligament (ACL) injuries by limiting the input field-of-view to the intercondylar region in order to maximize the performance. The study also detailed how adding contextual information from nearby image slices can improve the accuracy of networks. For training and testing, 260 participants were evenly divided into total ACL tear and normal cases using coronal PD non-fat suppressed images. The proposed model detected ACL tears with 96.7% accuracy.

Yan et al. (2023) proposed a federated self-supervised learning framework that ensures privacy and employs masked image modeling to train models cooperatively using decentralized data as a self-supervised task. This approach significantly outperforms the state-of-the-art ImageNet supervised pretraining baseline model, particularly under conditions of significant data heterogeneity. Additionally, it demonstrates effective learning with limited labeled data and generalizes well to out-of-distribution data. They trained and tested their approach on the Retina Dataset, Dermatology Dataset, COVID-FL Dataset, and Skin-FL Dataset, achieving accuracies of 81.94% on the Retina Dataset, 93.55% on the Dermatology Dataset, and 95.77% on the COVID-FL Dataset. In this research, Lei et al. (2023) introduced a framework called FedDAvT, which stands for multi-site federated domain adaptation via Transformer. This framework aims to protect data privacy and reduce data differences. They use a Transformer network as the main component to identify connections between different regions of interest in brain data, capturing detailed brain information. In order to adjust the model for both the source and target domains, they aligned the self-attention maps by utilizing the mean squared error. The analyzed datasets comprise the Australian Imaging, Biomarker and Lifestyle Flagship Study of Ageing (AIBL), Alzheimer’s Disease Neuroimaging Initiative (ADNI), and AI4AD data. The results indicate that the proposed FedDAvT method is highly effective, with accuracy rates of 69.51, 88.75, and 69.88% achieved in the AD vs. NC, MCI vs. NC, and AD vs. MCI two-way classification tasks, respectively. In this context, AD refers to Alzheimer’s Disease, NC refers to Normal Control, and MCI refers to Mild Cognitive Impairment.

Peta and Koppu (2023) implemented Extended ElGamal Image Encryption (E-EIE) to encrypt medical images using keys optimized through the Improved Sand Cat Swarm Optimization (I-SCSO) algorithm. These encrypted images are securely stored and transmitted using the Federated Learning Flower (FLF) framework, ensuring high levels of data security. For disease classification, the encrypted data is decrypted and processed using the Convolutional Capsule Twin Attention Tuna Optimal Network (C2T2Net), where further optimization is achieved by fine-tuning parameters using Chaotic Tuna Swarm Optimization (CTSO) technique. The model was evaluated using BreakHis dataset, attaining a classification accuracy of 95.68%.

Wu et al. (2022) proposed Training Efficient Federated Active Learning (TEFAL) and Labeling Efficient Federated Active Learning (LEFAL) where LEFAL utilized a hybrid sampling model for both diversity and uncertainty to enhance labeling efficiency in a task-agnostic context. TEFAL improved client-side performance by employing a discriminator mechanism to evaluate the informativeness of clients. These techniques collectively enhanced the efficiency and performance of federated active learning. The model was tested on the Hyper-Kvasir and CC-CCII datasets, resulting an accuracy of 84 and 97.6%, respectively.

Ouyang et al. (2022) present SSL-ALPNet, a novel self- supervised Few-Shot Segmentation (FSS) framework for medical images that does not require annotations during training. This technique generates supervision signals by utilizing pseudo-labels based on super pixels. To further improve segmentation accuracy, they suggest integrating an adaptive local prototype pooling module into prototype networks. Using the abdominal CT and abdominal T2-SPIR MRI datasets, they achieved dice scores of 75.91 and 80.16, respectively. Qiu et al. (2023) propose a novel approach for developing a Federated Semi-Supervised Learning (FSSL) model across distributed medical image domains. They introduce a federated pseudo-labeling strategy for unlabeled clients; leveraging embedded knowledge learned from labeled clients. This approach effectively addresses the issue of insufficient annotations in unlabelled clients, leading to a cost-efficient and streamlined solution for medical image analysis. The Dice scores obtained for the fundus imaging and prostate MRI segmentation tasks are 89.23 and 91.95%, respectively.

Zeng et al. (2022) introduced an approach called gradient matching federated domain adaptation (GM-FedDA) for classifying brain images. The goal of this strategy is to minimize differences between domains and train strong local federated models for specific target sites. The method comprises of two primary stages: the pretraining stage, which introduces a strategy called one-common-source adversarial domain adaptation (OCS-ADA), and the fine-tuning stage, which employs a method called gradient matching federated (GM-Fed) fine-tuning to update the local federated models pretrained with the OCS-ADA strategy. They achieved an AUC score of 90.93 on the SCZ dataset and 76.63 on the MDD dataset. Jiang et al. (2022) describe a novel multi-learner methodology for identifying various medical images. The proposed model integrated a task-learner, metric-learner, and autoencoder, trained using transfer and meta-learning to enhance adaptability in few-shot scenarios. In a 3-way 5-shot classification setting, the model achieved accuracies of 72% on the Blood dataset, 75% on the Pathology dataset, and 48% on the Chest dataset.

Singh et al. (2021) proposed few-shot learning framework named MetaMed that used meta-learning techniques and integrated data augmentation strategies such as CutOut, MixUp, and CutMix to improve generalization and model robustness. Comparative analysis showed that meta-learning consistently outperformed traditional transfer learning across differents datasets evaluated. sMetaMed achieved test accuracies of 74% on BreakHis, 65.41% on ISIC 2018, and 80% on the Pap Smear dataset under a 3-way 5-shot configuration.

Chen et al. (2023) proposed the Dynamic Federated Meta-Learning (DFML) approach, aimed to enhance rare disease prediction. They developed Inaccuracy Focused Meta-Learning (IFML) technique, which adjusted attention across tasks based on the performance of local learners. To improve the federated learning process, a dynamic, accuracy-driven client selection and model fusion strategy was implemented resulting s91 and 95% accuracy in 5-shot classification tasks on the Arrhythmia and FECG datasets, respectively.

Nurgazin and Tu (2023) analysed the application of Vision Transformers (ViTs) combining with few-shot learning algorithms such as ProtoNet, MatchingNet, and Reptile. In a 3-way 5-shot task, the models attained accuracies of 76% on BreakHis, 75% on ISIC 2018, and 89% on Pap Smear datasets.

Khandelwal and Yushkevich (2020) proposed a model-agnostic meta-learning framework to enhance domain generalization for biomedical imaging. This approach was analysed using a sCT vertebrae segmentation experiments conducted across three datasets, which included samples from both healthy and infected cases. In addition, they utilized few-shot learning, which involves training the generalized model using only a few number of samples from an unseen domain. This allows the model to rapidly adapt to new and unforeseen data distributions. They achieved a dice score of 87.85 when they trained the model on CSI challenge, xVertSeg segmentation challenge, VerSe MICCAI segmentation challenge 2020 datasets and tested it on an unseen domain. Chen et al. (2023), introduced MetaLR, an LR tuner that utilized meta-learning to enable the automatic co-adaptation of multiple layers in response to downstream tasks, taking into account their transferability across different domains. MetaLR dynamically adjusts the learning rates for different layers in an online fashion, ensuring that highly transferable layers retain their medical representation abilities and promoting active adaptation of less transferable layers to new domains. They achieved accuracies of 94, 87, and 96% on POCUS, BUSI, and Chest X-ray datasets, respectively, and a dice score of 94.2 on the LiTS dataset. Islam et al. (2023) suggested Federated Learning (FL) to tackle the problem of centralized data collecting in the context of brain tumor diagnosis from MRI scans. At first, many CNN models were trained using the MRI data. The three most successful CNN models were then chosen to create various versions of ensemble classifiers. Following that, the FL model was built using the ensemble architecture and trained utilizing model weights from the local models without exchanging the customers’ data (MRI images) through the FL technique. The researchers obtained a precision rate of 91.05% by utilizing federated learning (FL) to detect brain tumors. Li et al. (2022) proposed a novel Domain Generalization (DG) scheme using episodic training with task augmentation for medical imaging classification. Leveraging meta-learning, they develop a paradigm of episodic training to facilitate knowledge transfer from simulated training tasks to real testing tasks in DG. Task augmentation is introduced to increase training task variety. Additionally, they employ a new meta-objective to regularize the deep embedding of training domains within the established learning framework achieving a mean accuracy of 91.77%.

Several studies have investigated the mid- to long-term outcomes of tibial plateau fractures (TPFs) treated with open reduction and internal fixation (ORIF), emphasizing the importance of early radiographic parameters in predicting clinical prognosis. Biz et al. (2019) conducted a retrospective study analysing mid-term radiographic and functional outcomes in patients with AO 41-B and 41-C TPFs treated with ORIF. Their findings showed that AO 41-C fractures were associated with worse clinical outcomes and a higher incidence of post-traumatic osteoarthritis (PTOA), particularly when postoperative malalignment or articular step-off was present. Functional recovery, assessed using KOOS, AKSS, and SF-36 scores, was significantly influenced by patient age and BMI. These results align with other studies that report early radiographic features, such as tibial alignment and joint surface congruity, as predictors of pain and long-term functional limitations. Belluzzi et al. (2019) explored the role of different joint tissues—cartilage, synovial membrane, meniscus, and infrapatellar fat pad (IFP)—in promoting synovitis, a key pathological feature of OA. Using conditioned media from these tissues to stimulate fibroblast-like synoviocytes (K4IM cells), they observed elevated levels of IL-6, IL-8, and CCL2 across all tissue types. However, only synovium-derived media induced significant upregulation of inflammatory genes such as IL-6, CXCL8, and MMP-10, highlighting the synovial membrane’s dominant role in driving synovial inflammation. These findings complement studies like Biz C et al., which emphasized the impact of joint structural damage, such as articular step-off and malalignment, on long-term outcomes and inflammation in TPF cases. Together, these works reinforce the notion that both structural integrity and tissue-level inflammatory responses critically influence OA progression and post-traumatic recovery (Table 1).

**Table 1:** Summary of different studies on knee injury diagnosis.

Author	Dataset	Method	Result
Tsai et al. (2020)	MRNet, KneeMRI	Efficiently-Layered Network (ELNet)	ROC-AUC score for Meniscus Tear-0.904, ACL Tear-0.96 and Abnormality-0.941
Azcona et al. (2020)	MRNet	MRNet design replaced with the AlexNet model with a Pretrained ResNet model (18, 50, 152).	Achieved combined AUC score of 0.934
Dai et al. (2021)	MRNet, PGT	Incorporated the self-attention mechanism into the multi-modal fusion technique through the use of transformers.	ROC-AUC score for Meniscus Tear-0.952, ACL Tear-0.981 and Abnormality-0.976
Joshi and Suganthi (2022)	MRNet	Three-layered compact parallel deep convolutional neural network (CPDCNN) for the detection of ACL tears.	Accuracy-96.6%
Yan et al. (2023)	MRNet	Adversarial data augmentation with MRNet Architecture	Achieved combined AUC score of 0.8953%
Al Ghothani and Zhang (2023)	MRNet	Decision fusion, feature fusion with Convolution Block Attention Module (CBAM) attention	combined AUCscore-0.925%
Kara and Hardalaç (2021)	MRNet	Adversarial data augmentation with MRNet Architecture	ROC-AUC score for Meniscus Tear-74.5, ACL Tear-0.86 and Abnormality-0.86
Bien et al. (2018)	MRNet	MRNet, a deep learning model, to automatically analyze knee MRI tests.	ROC-AUC score for Meniscus Tear-0.847, ACL Tear-0.965 and Abnormality-0.937
Hung et al. (2023)	MRNet	Enhanced YOLOv4 model, which used Darknet-53 as its foundation	Accuracy-78.8%
Li et al. (2023)	MRNet, MRI-ACL	For detecting ACL tear in Knee MRI they proposed using a 3D weighted multi-view convolutional neural network.	AUC score achieved was 92.86% for ACL Tear
Belton et al. (2021)	MRNet	MPFuseNet network, in which it makes use of a multi-view, pre-trained CNN with a spatial attention block.	Achieved AUC score of 0.957 for Abnormality, 0.977 for ACL, and 0.831 for Meniscus Tear
Javed Awan et al. (2021)	KneeMRI	Modified 14-layer CNN architecture, to prevent over- fitting and maintain efficiency while detecting ACL tears.	Achieved Accuracy of 92%
Li et al. (2022)	Private dataset	Meniscus tears on MRI images were identified and predicted using a Mask R–CNN model in this work.	Accuracy for healthy, torn and degenerated menisci was 87.50, 86.96, and 84.78%, respectively.
Dunnhofer et al. (2022)	MRNet, FastMRI+	MRPynet architecture aimed at improving small object identification.	Achieved an accuracy of 88.6% on ACL tear. On Meniscus tear achieved an accuracy of 77.8%.
Chang et al. (2019)	Private Dataset	CNN with dynamically sampled randomly cropped patches to identify ACL Tear	Accuracy of 96.7% for ACL Tear
Yan et al. (2023)	Retina, Dermatology, COVID-FL, Skin-FL	Federated self- supervised learning framework that ensures privacy	Achieved an accuracy of 81.94% on Retina, 93.55% on Derm, 95.77% on COVID-FL.
Lei et al. (2023)	ADNI, AIBL, AI4AD	FedDAvT to protect data privacy and re duce data differences.	Achieved accuracy rates of 88.75, 69.51, and 69.88% on the AD vs. NC, MCI vs. NC, and AD vs. MCI classification tasks
Peta and Koppu (2023)	BreakHis Dataset	Federated learning flower and C2T2Net model for disease classification	Achieved an accuracy of 95.68%.
Wu et al. (2022)	Hyper-Kvasir dataset, CC-CCII dataset	LEFAL with the goal of improving data efficiency. TEFELto improve client efficiency	Achieved an accuracy of 97.6% on CC-CCII dataset and 84% accu racy on Hyper-Kvasir dataset
Ouyang et al. (2022)	abdominal CT, abdominal T2- SPIR MRI, cardiac bSSFP MRI	SSL-ALPNet, a novel FSS framework for medical images	Achieved a dice score of 75.91, 80.16% for abdominal CT, abdominal T2-SPIR MRI respectively
Qiu et al. (2023)	RIMONE-r3, Drishti-GS, NCI- ISBI, I2CVB, PROMISE12	FSSL model across distributed medical image do mains.	Achieved Dice scores of 89.23 and 91.95% on fundus image and prostate MRI segmentation tasks, respectively.
Zeng et al. (2022)	SCZ Dataset, MDD Dataset	GM-FedDA method for brain image classification. The method consists of two main stages: the pretraining stage, and the fine- tuning stage.	Achieved an AUC Score of 90.93% on SCZ dataset and AUC Score of 76.63% on MDD Dataset.
Jiang et al. (2022)	BLOOD, PATHOLOGY, CHEST datasets	Combined meta-learning, transfer learning, and metric learning	Achieved an accuracy of 72, 75, 48% in 3 way 5 shot Classification task on Blood, Pathology and chest datasets, respectively.
Singh et al. (2021)	BreakHis, ISIC 2018, pap Smear	MetaMed approach that uses meta- learning to handle medical image classification.	Achieved an accuracy of 74, 65.41, 80% in 3 way 5 shot classi fiication task
Chen et al. (2023)	Arrhythmia dataset, FECG dataset	To improve the prediction of rare diseases, they present a Dynamic Federated Meta-Learning (DFML) approach in this study.	Achieved an accuracy of 91, 95%, in 5 shot classification task.
Nurgazin and Tu (2023)	BreakHis, ISIC 2018, pap Smear	This study explores the application of ViT in few-shot learning scenarios for medical image analysis.	Achieved an accuracy of 76, 75 and 89%, in 3 way 5 shot classification task.
Khandelwal and Yushkevich (2020)	CSI, xVertSeg, VerSe MICCAI	This work adapts a domain generalization method based on a model- agnostic meta-learning framework to biomedical imaging.	Achieved a dice score of 87.85%
Chen et al. (2023)	POCUS, BUSI, Chest X-ray, LiTS	meta- learning-based LR tuner, named MetaLR that learns appropriate LRs for different layers, preventing highly transferable layers from forgetting their medical representation.	Achieved an accuracy of 94, 87, 96% on POCUS, BUSI, Chest X-ray, respectively, and also an achieved a dice score of 94.2% on LiTS dataset.
Islam et al. (2023)	UK Data Service	This paper, proposes addressing the centralized data collection issue by applying FL to brain tumor identification from MRI images.	Achieved an accuracy of 91.05% using FL for detecting brain tumors
Li et al. (2022)	VGH, NKI, IHC and NCH, LiTS	DG scheme using episodic training with task augmentation for medical imaging classification.	Achieved a mean accuracy of 91.77%

## 3 Proposed architecture

**Figure 1:**
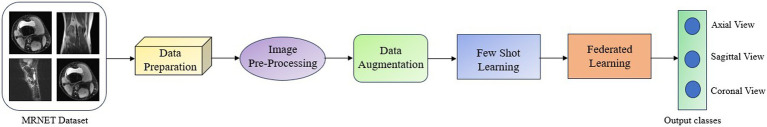
Proposed architecture for federated knee injury diagnosis using few shot learning.

The proposed Federated Few Shot Knee Injury Diagnosis system comprises the following modules: Dataset Description, Data Preprocessing, Image Preprocessing, Data Augmentation, Few Shot Learning and Federated Learning and the flow is shown in Figure 1.

### 3.1 Dataset description

The Stanford University Medical Center’s MRNet dataset (Bien et al., 2018; MRNet dataset, 2019), was assembled from knee MRI images performed between January 2001 and December 2012. The dataset consists of 1,370 samples, with 1,104 abnormal cases out of which 508 are meniscus tears and 319 are ACL tears, which were acquired by manual extraction from clinical reports.

**Figure 2:**
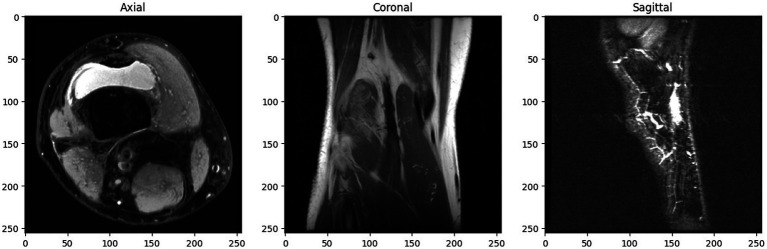
Multi-view knee MRI slice of MRNet dataset.

The dataset is splitted into three separate sets: 1,130 exams from 1,088 patients comprise the training set; 120 exams from 111 patients make up the validation set; and 120 exams from 113 patients make up the hidden test set. With three different image views—Coronal T1 weighed 3D MRI, Sagittal T2 weighted 3D MRI, and Axial PD weighted 3D MRI—each case. Figure 2 depicts a slice of a patient’s knee MRI, exhibiting three distinct image views: axial, coronal, and sagittal.

### 3.2 Data preprocessing

The MRNet dataset also contains three CSV files, each dedicated to abnormality, ACL tear, and meniscus tear, respectively. Within each file, entries are labeled with a ‘1’ to indicate the presence of the corresponding condition and ‘0’ to signify its absence. During pre-processing these labels were combined, resulting in combinations like (0, 0, 0), (1, 0, 0), (1, 0, 1), (1, 1, 0), and (1, 1, 1).

1. (0, 0, 0): This combination indicates the absence of all three conditions - abnormalities, ACL tears, and meniscus tears. This class is denoted by label ‘0’.
2. (1, 0, 0): This combination signifies the presence of an abnormality but no ACL tear or meniscus tear. This class is denoted by label ‘1’.
3. (1, 0, 1): Here, the presence of an abnormality and a meniscus tear is indicated, but there is no ACL tear. This class is denoted by label ‘2’.
4. (1, 1, 0): This combination denotes the presence of an abnormality and an ACL tear, but no meniscus tear. This class is denoted by label ‘3’.
5. (1, 1, 1): Finally, this combination indicates the presence of all three abnormalities - an abnormality, an ACL tear, and a meniscus tear. This class is denoted by label ‘4’.

**Figure 3:**
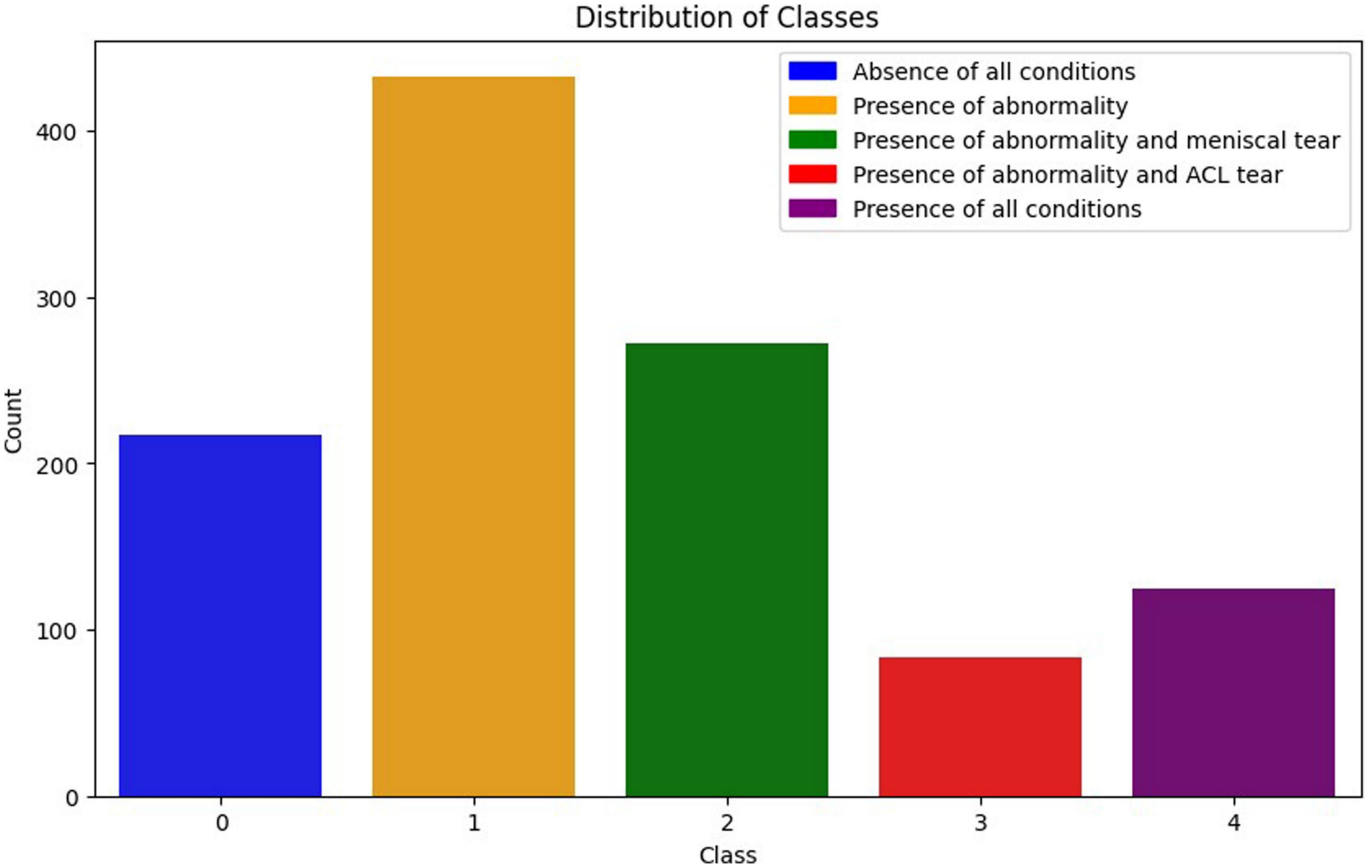
Distribution of the classes.

Each combination represents various scenarios of injuries in patients. This data organization facilitates multi-label classification, allowing a model to simultaneously learn and predict multiple conditions. Figure 3 illustrates the class distribution imbalance within the dataset. The figure shows the varying quantities of images among different classes, with Class 1 exhibiting the highest count and Class 3 the lowest.

### 3.3 Image preprocessing

The number of slices in the Knee MRI of a patient in the MRNet dataset ranges from 17 to 61. To overcome this issue, a linear interpolation procedure is employed to produce a constant number of slices for examination. By calculating the weighted average of adjacent slices, new slices are created between the original ones through the process of linear interpolation. This method assigns 80% of the weight to the subsequent slice and the remaining 20% to the preceding slice, which results in the creation of 15 new slices. This method effectively standardizes the number of slices across the dataset. To ensure compatibility with the expected input format of the model, a grayscale channel is repeated and the image pixel values are normalized.

### 3.4 Data augmentation

Data augmentation is a technique of using various modifications on data samples to artificially enlarge a dataset. Data augmentation helps avoid over-fitting and increases the model’s resilience to unknown data by adding variations to the input data while maintaining the ground truth labels. In this study, data augmentation is utilized not to generate entirely new images or slices but to augment the existing training data, enabling the model to learn more robust features and patterns. For every training image slice, the following data augmentation methods were applied.

1. Resizing: Image slice sized 256 × 256 pixel resized to 224 × 224 pixel size uniformly to each image slice.
2. Horizontal flip: This technique involves mirroring the image along its horizontal axis to produce a new version in which the object is perceived to be facing the other way. 50% random flip strategy is implemented; therefore there is a 50% chance that each image slice will be flipped horizontally during training.
3. Vertical Flip: A vertical flip mirrors the image along the vertical axis. This can be achieved mathematically by transforming the image matrix using a vertical reflection transformation. For vertical flips, 50% random flip is applied, just like in the horizontal flip.
4. Random Rotation: Rotations cause variations in object orientation within the plane. The rotations between −45 and 45 degrees are used in this paper. By transforming the image coordinates using a random rotation matrix, this is accomplished. Rotations introduce variations in the possible positions of objects within the image slice which aids in the model’s ability to recognize image in any pose.
5. Affine Transformations: This paper utilizes random affine transformations for scale, rotation, and translation. Scale variations train the model to recognize objects regardless of size within the image. Rotation within the affine transformation results non-uniform angles and potential shearing. Translation results random shifts in all directions to generate svariations in object position.

To enhance the diversity of image slices in the training dataset and strengthen the model’s generalization ability, the above mentioned data augmentation techniques have been utilized.

### 3.5 Few shot learning

Few-shot learning is a deep learning approach designed to enable models to make accurate predictions or classifications using only a small number of samples per class. Unlike traditional deep learning methods that rely on large-scale labeled datasets, few-shot learning is effective with minimal annotated data, making it particularly useful in scenarios where data collection and labeling are challenging or costly. In this framework, the “support set” refers to a limited set of labeled examples used during training, while “support labels” denote their corresponding class labels. The “query set” consists of unlabelled samples for which the model must generate predictions. The terminology “n-way k-shot” is commonly used in few-shot learning, where “n” indicates the number of distinct classes and “k” represents the number of samples per class in the support set. A 3-way 5-shot classification includes five training examples for each three classes, and the model is tasked with identifying the correct class for each query. Few-shot learning has the ability to quickly adjust to new tasks or domains with little labeled data, which makes it extremely effective in situations where data is lesss.

**Figure 4:**
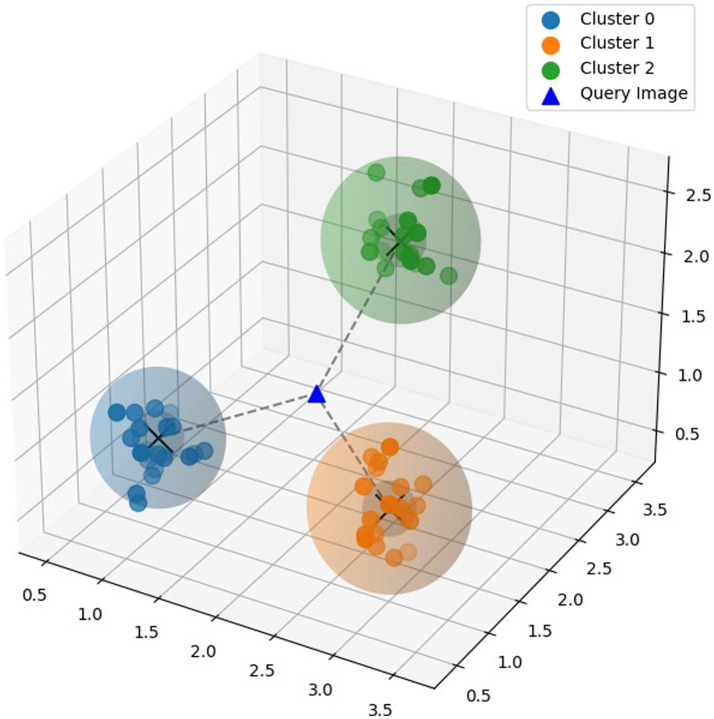
Prototypical model.

Metric learning learns similarity measure between data points and extracts a distance metric directly from the data, by comparing the similarity between samples. Metric learning can also handle high-dimensional data efficiently, making it suitable for tasks involving complex feature spaces. Prototypical Network (Snell et al., 2017) is a few shot learning model based on metric learning. The Prototypical Network aims to learn a metric space where data points belonging to the same class are separated from points belonging to other classes. Prototypes are built initially by calculating the mean of the embedding of the instances that make up each class. The model then uses a distance metric, such as the Euclidean distance, to determine how far a given query instance is from each class prototype as shown in Figure 4. It then allocates the query image to the class in the embedding space that has the closest prototype. This procedure is carried out once more for every query instance, enabling effective classification. Prototypical Network has the capacity to learn a metric space that enables effective classification even with a small amount of training data, because of which it can generalize well to previously undiscovered classes.

Given:

$S=\{(x_{1},y_{1}),\ldots \ldots .,(x_{N},y_{N})\}$ , where *N* is the number of labeled examples in the support set.

$X_{i}\in R^{D}$ is the *D*-dimensional feature vector of example i .

$Y_{i}\in \{1,\ldots ..,K\}$ , is the corresponding label of example *i*, where *K* is the total number of classes.

$S_{k}$ denotes the set of examples labeled with class *k*.

The prototypical network formula for calculating the prototype of class *k*, denoted as $\;C_{k}$ , as mentioned in Equation 1:


(1) $$\;C_{k}=\frac{1}{|S_{k}|}\;\sum_{(x_{i},y_{i})\in S_{k}}f_{\varphi }(x_{i})$$

where $\;C_{k}$ represents the prototype of class *k*, which is the mean feature vector of all the support examples belonging to class *k*, $|S_{k}|$ denotes the number of examples in the support set labeled with class *k*. $\sum_{(x_{i},y_{i})\in S_{k}}f_{\varphi }(x_{i})\;$ signifies summation over all examples $\left(x_{i},y_{i}\right)$ in the support set $S_{k}$ , i.e., all examples belonging to class *k* and $\;f_{\varphi }(x_{i})$ is the feature representation of example $x_{i}$ .

### 3.6 Federated learning

**Figure 5:**
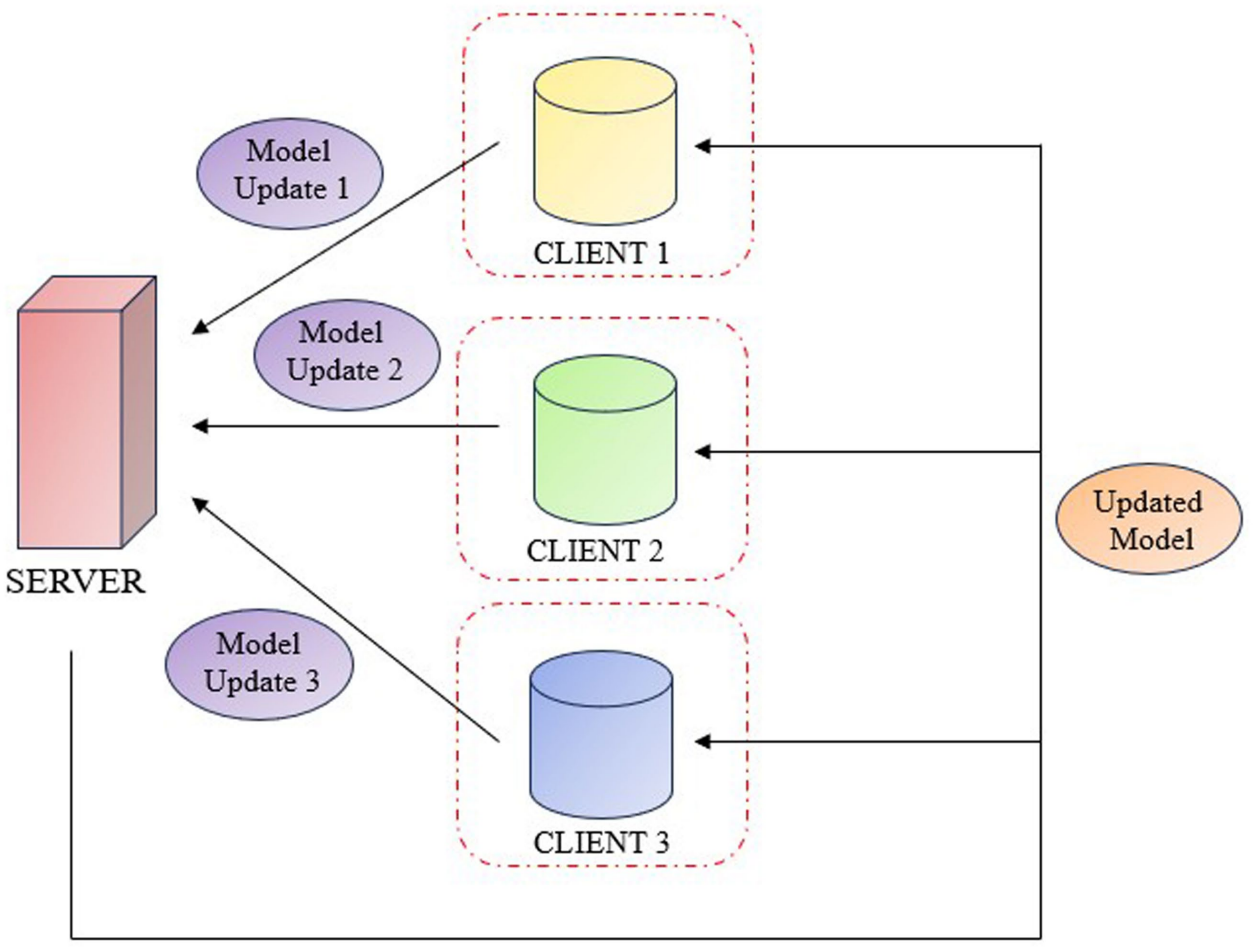
Federated learning system.

Federated learning is a cooperative machine learning technique in which clients train a model with a decentralized data. This allows hospitals or research centres, to work together to improve the precision of deep learning models for medical diagnosis without exchanging patient information. Each client can train its local model and periodically updates the central system with the weights. The central system aggregates these weights and refines the global model, integrating the results from all the clients. Federated Learning enables collaborative training across institutions while preserving data privacy. FL is categorized into horizontal, vertical, and federated transfer learning. Horizontal FL applies when datasets have similar features but different samples, such as MRI scans from various hospitals, and allows secure model training without sharing raw data. Vertical FL is used when datasets involve the same individuals but different features, enabling collaboration between institutions like hospitals and genomics labs. Federated Transfer Learning is suitable for institutions with differing samples and features, using transfer learning to combine domain knowledge. In the proposed work horizontal FL is adopted to aggregate knee MRI data from multiple sources with a shared feature space. This approach improves diagnostic accuracy while maintaining data confidentiality and supporting scalability across medical centers (Figure 5).

## 4 Experimental results

### 4.1 Implementation details

This section outlines the proposed system, detailing the network architecture, training process, fine-tuning, and evaluation. Each stage is designed to enhance the model’s performance and adaptability for few-shot knee injury diagnosis. The experimentation of the proposed model was analysed on a 64-bit Windows 10 operating system, implementation were carried out using Python notebook. The hardware configuration used included an Intel® Xeon® Gold 6,230 CPU @ 2.10GHz processor, 64 GB of RAM, an NVIDIA Quadro RTX 5000 GPU with 16 GB of memory, and a 2 TB hard disk.

#### 4.1.1 Network architecture

**Figure 6:**
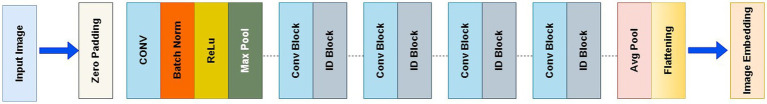
ResNet50 backbone architecture.

The proposed work used pre-trained 3D ResNet-50 model integrated as the CNN backbone to extract image features. MedicalNet (Chen et al., 2019) was pre-trained on 23 diverse medical datasets utilizes extensive knowledge from various medical images, improving the model’s ability to generalize to new data, and it reduces training time and computational requirements compared to training from scratch especially beneficial for small medical imaging datasets (Figure 6).

#### 4.1.2 Training stage

The training stage of Centralized and Federated Few Shot System makes use of the episodic few-shot learning method using Prototypical Network. In this phase there are 500 tasks in every epoch and each task is a 3-way 5-shot classification scenario. A 2-way 8-shot setup across 100 tasks per epoch is used for validation. The training protocol consists of an initial learning rate of 0.01 enabled by the Stochastic Gradient Descent (SGD) optimizer with weight decay set to 5e-4 and momentum set to 0.9, among other parameters. The Cross Entropy Loss function is used to measure performance. Furthermore, a MultiStep LR scheduler is used to adaptively modify the learning rate during training. This scheduler has milestones set at tasks 120 and 160 and a gamma value of 0.1. The task sampler used in this study is available in the open source EasyFSL library (Bennequin, 2023).

During the implementation of FL, the training data was divided between two clients. Each client completed the training phase of the model locally and sent the weights of the model with best validation accuracy to the global model for weighted averaging. After averaging the updated weights were sent back to the clients to continue with the fine-tuning process locally. During the implementation of the Centralized Few shot system, the model with the highest validation accuracy is chosen. In the training phase, the idea of few-shot learning is applied to acquire initial knowledge with a small amount of data.

#### 4.1.3 Fine-tuning stage

Stage using an episodic few shot approach, the Prototypical Network is fine-tuned in this phase locally by the model in both Centralized and Federated Few Shot System. During this stage, new classes different from the ones used in the training phase are used. The weights from the training phase are employed in this phase. A 2-way 5-shot setup with 100 tasks per epoch for validation and a 3-way 5-shot setup with 500 tasks per epoch for training are used in this fine-tuning process. It is validated using the same loss function, optimizer, and other parameters as the training phase. Through this process of fine-tuning, the model is able to better adapt to completely new tasks and classes by making use of its initial learning experience, which was obtained from a small number of examples in the training phase.

#### 4.1.4 Evaluation

The model with the best validation accuracy at the end of the fine-tuning phase is chosen for final testing. This model is evaluated using performance metrics which include Accuracy Score, F1 score, Precision, and Recall, in a 2-way 8-shot setting. These metrics offer a comprehensive comprehension of the model’s efficacy in classification tasks, guaranteeing a comprehensive evaluation of its performance concerning multiple dimensions such as total accuracy, equilibrium between precision and recall, and its ability to generalize to novel data.

### 4.2 Result analysis

From Table 2 it can be inferred that in a centralized few shot system, the model performed particularly well in the axial plane, then in the sagittal plane, and lastly in the coronal plane. This suggests a general difficulty in identifying knee injuries using coronal plane data.

**Table 2:** Results obtained using centralized few shot system.

Plane	Training Class	Testing Class	Accuracy (%)	F1 Score	Precision	Recall
Axial	(1, 2, 4)	(0, 3)	85.3	0.852	0.853	0.852
Axial	(1, 3, 4)	(0, 2)	74.9	0.749	0.749	0.749
Axial	(1, 2, 3)	(0, 4)	79.4	0.790	0.808	0.794
Axial	(0, 1, 4)	(2, 3)	69.1	0.691	0.691	0.691
Axial	(0, 1, 3)	(2, 4)	69.9	0.698	0.699	0.698
Axial	(0, 1, 2)	(3, 4)	60.1	0.600	0.601	0.600
Sagittal	(1, 2, 4)	(0, 3)	74.0	0.740	0.741	0.740
Sagittal	(1, 3, 4)	(0, 2)	82.0	0.819	0.820	0.820
Sagittal	(1, 2, 3)	(0, 4)	82.1	0.819	0.830	0.820
Sagittal	(0, 1, 4)	(2, 3)	66.7	0.663	0.676	0.667
Sagittal	(0, 1, 3)	(2, 4)	56.9	0.559	0.576	0.569
Sagittal	(0, 1, 2)	(3, 4)	55.0	0.549	0.551	0.55
Coronal	(1, 2, 4)	(0, 3)	53.8	0.536	0.539	0.538
Coronal	(1, 3, 4)	(0, 2)	61.9	0.618	0.619	0.618
Coronal	(1, 2, 3)	(0, 4)	65.1	0.651	0.652	0.651
Coronal	(0, 1, 4)	(2, 3)	71.0	0.709	0.712	0.710
Coronal	(0, 1, 3)	(2, 4)	58.2	0.580	0.583	0.582
Coronal	(0, 1, 2)	(3, 4)	51.5	0.515	0.516	0.516

Moreover, the highest accuracy was consistently achieved when testing class was class 0 with a combination between classes 2, 3, or 4. This shows how well the model can distinguish between classes that represent no abnormality, meniscus tears, ACL tears, and the classes containing combinations of injuries. When testing between Classes (2, 3), (2, 4), or (3, 4), however, performance declines, indicating that it may be difficult to differentiate between classes containing combinations of injuries. Because these classes are similar, the testing class with the lowest accuracy across all planes was (3, 4).

With training classes (1, 2, 4) and testing classes (0, 3), the model performed best when trained and tested on the axial plane. This resulted in an accuracy of 85.3% and an F1 Score of 0.852. Similarly, with an accuracy of 82.1% and an F1 Score of 0.819 on the sagittal plane, the model performed best when trained and tested using the training classes (1, 2, 3) and testing classes (0, 4). With an accuracy of 82% and an F1 Score of 0.819, it also performed well when trained and tested using the training classes (1, 3, 4) and testing classes (0, 2). Despite the overall superior performance of the axial plane, the sagittal plane showed greater consistency in distinguishing Class 0 from higher-numbered injury classes, indicating that sagittal features may capture more distinguishable cues for detecting the absence of pathology across diverse training configurations.

With training and testing classes (0, 1, 4) and testing classes (2, 3), the model performed best on the coronal plane, obtaining an accuracy of 71% and an F1 Score of 0.709. This implies that when attempting to distinguish between an ACL tear and a meniscus tear, the coronal plane is more useful.

The training and testing classes in the centralized few-shot system that showed the best accuracy in each plane was then used in a federated few-shot system with two clients. Table 3 demonstrates that accuracy of classes in axial plane decreased just 2 to 83%, while in sagittal plane it increased to 83.9%. While in coronal plane it only slightly decreased 5 to 65.1%.

**Table 3:** Results obtained using federated few shot system.

Plane	Training class	Testing class	Accuracy (%)	F1 Score	Precision	Recall
Axial	(1, 2, 4)	(0, 3)	83.0	0.828	0.842	0.83
Sagittal	(1, 2, 3)	(0, 4)	83.9	0.839	0.84	0.839
Coronal	(0, 1, 4)	(2, 3)	65.1	0.649	0.655	0.651

It is significant that the model’s overall performance stayed similar across the plane and that using a federated system in place of a centralized one does not notably affect the model’s performance. Therefore, through Federated few shot system we can ensure that sensitive Knee MRI data is protected because it maintains data privacy by storing information locally on devices.

A comparison of several backbone architectures performances in a centralized few-shot learning system is shown in Table 4, with an emphasis on the axial, sagittal, and coronal planes. Pre-trained 3DResNet10, 3DResNet18, 3DResNet34, and 3DResNet50 are among the backbone architectures that were taken into consideration for evaluation; they were all derived from MedicalNet (Chen et al., 2019). While ResNet50 achieved the highest accuracy in the axial plane, its performance did not generalize across all views, suggesting that deeper architectures may be more plane-sensitive and potentially overfit to axial-specific spatial features.

**Table 4:** Performance comparison of different backbone in centralized system.

Plane	Backbone	Training class	Testing class	Accuracy (%)	F1 Score	Precision	Recall
Axial	Resnet 10	(1, 2, 4)	(0, 3)	74.1	0.738	0.75	0.74
Axial	Resnet 18	(1, 2, 4)	(0, 3)	80.4	0.804	0.806	0.804
Axial	Resnet 34	(1, 2, 4)	(0, 3)	0.767	0.767	0.767	0.767
Axial	Resnet 50	(1, 2, 4)	(0, 3)	85.3	0.852	0.853	0.852
Sagittal	Resnet 10	(1, 2, 3)	(0, 4)	90.4	0.904	0.905	0.904
Sagittal	Resnet 18	(1, 2, 3)	(0, 4)	82.1	0.82	0.821	0.82
Sagittal	Resnet 34	(1, 2, 3)	(0, 4)	85.2	0.851	0.857	0.852
Sagittal	Resnet 50	(1, 2, 3)	(0, 4)	82.1	0.819	0.83	0.82
Coronal	Resnet 10	(0, 1, 4)	(2, 3)	70.4	0.703	0.708	0.704
Coronal	Resnet 18	(0, 1, 4)	(2, 3)	66.1	0.661	0.662	0.661
Coronal	Resnet 34	(0, 1, 4)	(2, 3)	74.3	0.742	0.745	0.743
Coronal	Resnet 50	(0, 1, 4)	(2, 3)	71	0.709	0.712	0.71

A complex backbone such as ResNet50 appears to be most beneficial for the axial plane, which achieves the highest accuracy (85.3%) and F1 score (0.852). This implies that more detailed structural information may be present in the axial plane, necessitating a complex network for efficient feature extraction.

On the other hand, ResNet10 outperformed the other backbones in the sagittal plane, with an F1 score of 0.904 and an accuracy of 90.4%. This could be because a smaller, less complex network like ResNet10 is better at handling specific information, which the sagittal plane may prioritize capturing.

ResNet34 outperformed the other backbones under consideration in the coronal plane, achieving 74.3% accuracy with an F1 score of 0.742. This suggests that, in contrast to the extremes of ResNet 10 and ResNet50, the coronal plane might need a balance between complexity and information retention, which ResNet34 provides.

Table 5 demonstrates the significant improvements achieved by our approach in knee injury detection compared to prior methods. Our approach shows a notable increase in accuracy across all imaging planes. Specifically, our system attains 85.3% accuracy in the axial plane, nearly double the highest accuracy (45%) obtained by the ELNET model. In the sagittal and coronal planes, our model achieves 82.1 and 71.0% accuracy, respectively, greatly exceeding the best accuracies of 41.66 and 46.66% from previous methods. These results highlight our approach’s enhanced capability in accurately detecting knee injuries. Furthermore, the F1 Score, precision, and recall metrics further confirm our approach’s effectiveness. The proposed approach achieves F1 Scores of 0.852, 0.819, and 0.709 in the axial, sagittal, and coronal planes, respectively. These scores are substantially higher than those reported for other models, with the best F1 Scores from baseline models being 0.438 (ELNET, axial), 0.459 (MRNET- MRPyrNet, sagittal), and 0.452 (ELNET, coronal).

**Table 5:** Performance comparison of proposed system with previous knee injury detection approaches.

Author	Method	Plane	Accuracy (%)	F1 Score	Precision	Recall
Bien et al. (2018)	MRNET	Axial	42.5	0.387	0.554	0.425
		Sagittal	40.83	0.383	0.445	0.408
		Coronal	44.16	0.421	0.456	0.441
Tsai et al. (2020)	ELNET	Axial	45	0.438	0.590	0.45
		Sagittal	40	0.396	0.489	0.4
		Coronal	46.66	0.452	0.529	0.466
Dunnhofer et al. (2022)	MRNET-MRPyrNet	Axial	37.5	0.420	0.498	0.463
		Sagittal	41.66	0.459	0.515	0.508
		Coronal	30.83	0.348	0.435	0.381
Proposed	Proposed	Axial	85.3	0.852	0.853	0.852
		Sagittal	82.1	0.819	0.830	0.820
		Coronal	71.0	0.709	0.712	0.710

Additionally, the proposed method consistently surpasses others in precision and recall, indicating not only higher accuracy but also balanced performance in identifying true positive cases and minimizing false positives. For instance, both precision and recall for the axial plane are 0.852, significantly higher than the previous best precision of 0.590 (ELNET) and recall of 0.466 (ELNET) in the coronal plane. These baseline models were re-implemented and evaluated using multiclass classification across all five classes previously discussed in the paper. The results showed that the implementation of the Prototypical Network outperformed many baseline models. These metrics collectively shows that the proposed approach is more reliable and accurate for knee injury detection across various imaging planes.

**Figure 7:**
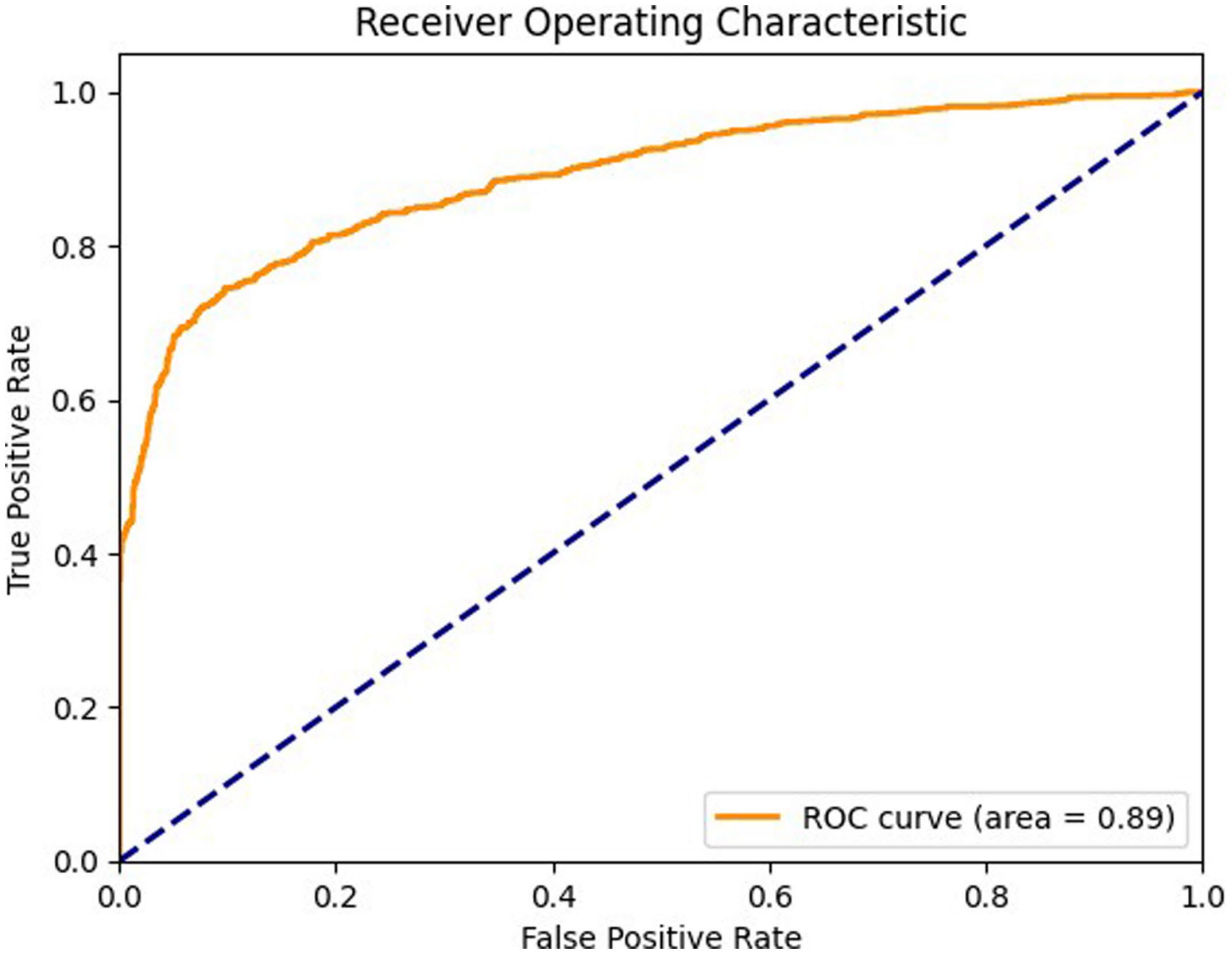
ROC curve for axial view.

**Figure 8:**
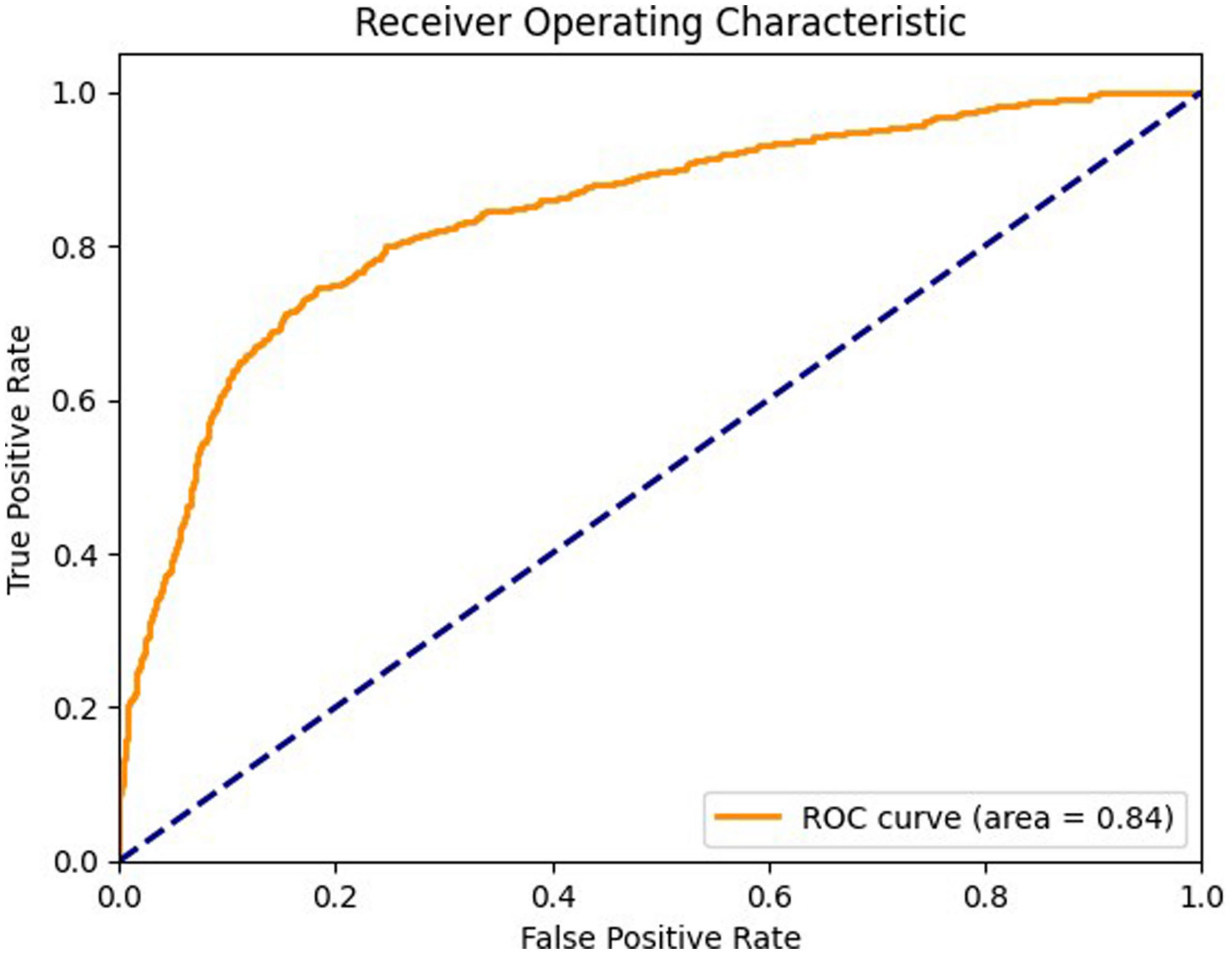
ROC curve for sagittal view.

Figure 7 shows ROC curve for the axial view, with an area under the curve (AUC) of 0.89 where the model effectively differentiates between positive and negative cases in the axial view. The curve maintains a high true positive rate (sensitivity) across various thresholds while keeping a low false positive rate (specificity). Figure 8 shows the ROC curve for the sagittal view with an AUC of 0.84. Although this AUC is slightly lower than the axial view, the model still demonstrates a reliable balance of sensitivity and specificity, accurately identifying true positive cases while minimizing false positives in the sagittal view.

**Figure 9:**
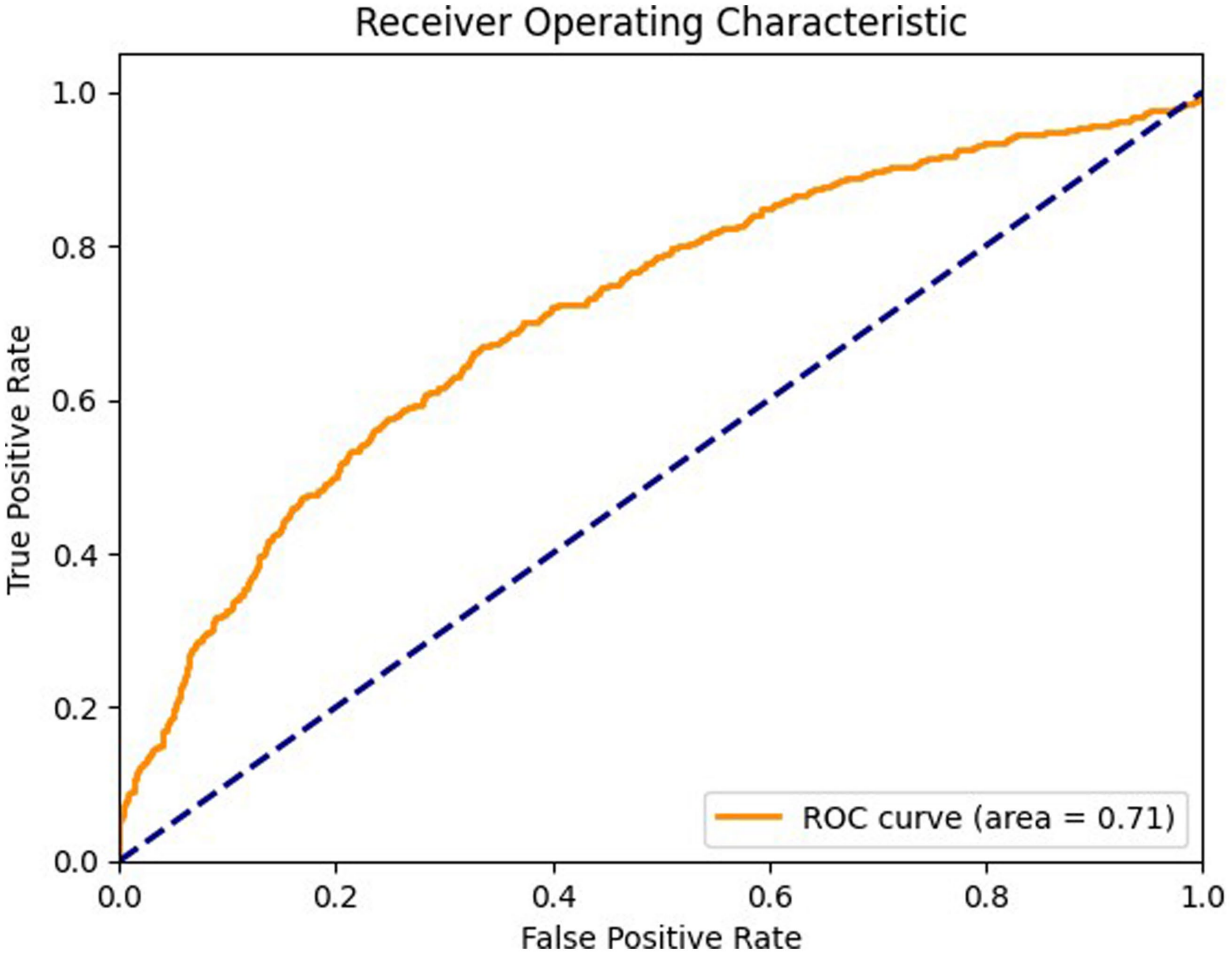
ROC curve for coronal view.

Figure 9 details the ROC curve for the coronal view, with an AUC of 0.71 where the model’s performance is moderate compared to the axial and sagittal views. The ROC curve for the coronal view shows that the model’s ability to differentiate between positive and negative cases is less effective, indicating a potential area for improvement. The model’s sensitivity and specificity are less balanced in this view, suggesting a need for optimization to enhance diagnostic accuracy.

**Figure 10:**
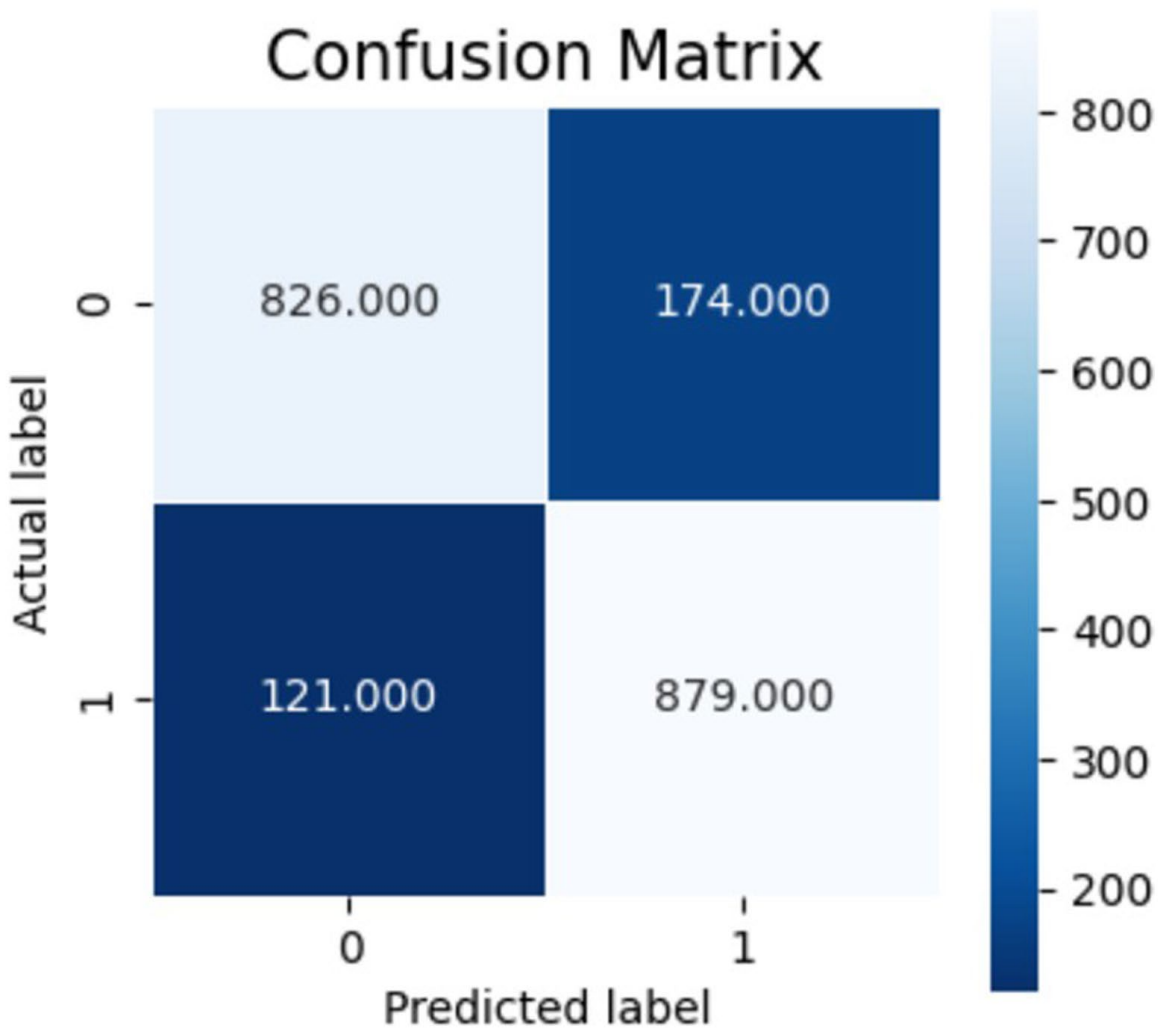
Confusion matrix for axial view.

**Figure 11:**
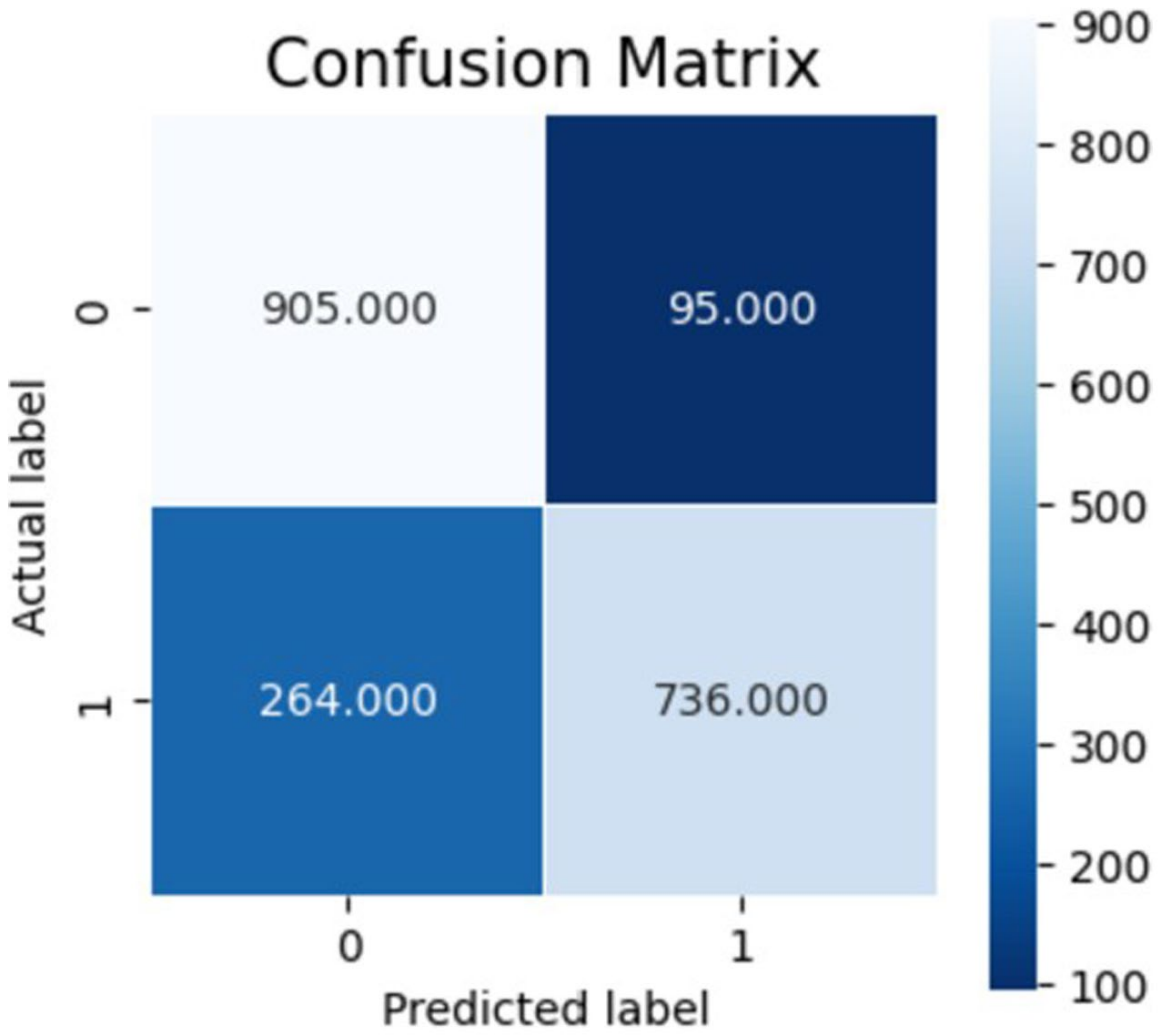
Confusion matrix for sagittal view.

**Figure 12:**
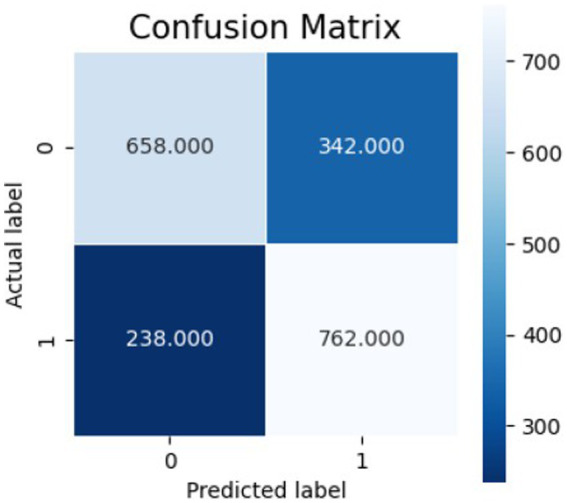
Confusion matrix for coronal view.

Figures 10–12 show the model’s performance across different testing classes and planes. In Figure 10 details high accuracy with a True Positive (TP) rate of 826 out of 1,000 for classes 0 and 3, a low False Positive (FP) rate of 121, and a False Negative (FN) count of 174, indicating actual class recognition and precision. Figure 11 shows the model’s exceptional accuracy for sagittal plane images with classes 0 and 4, achieving a TP count of 905, an FP count of 264, and an FN count of 95, alongside a high True Negative (TN) count of 736. Figure 12 highlights the model’s performance for coronal plane images with classes 2 and 3, achieving a TP count of 658, an FP count of 238, and an FN count of 342, with a high TN count of 762, showcasing its proficiency and resilience in classification.

## 5 Discussion

The proposed method’s accuracy in the axial plane, is 85.3%, and its F1 score is 0.852, significantly outperforming existing state of the art methods. The model’s performance is enhanced by combining a Prototypical Network with a ResNet-50 backbone, enabling effective few-shot learning for better results with limited training data.

The federated learning feature enhances robustness and generalizability by allowing decentralized training across institutions without sharing sensitive patient data, ensuring privacy and security. The model achieves 83.9% accuracy in the sagittal plane, 85.3% in the axial plane, and 82.1% in the sagittal plane, showing its versatility for knee injury diagnosis across imaging planes.

The proposed method exhibits a distinguished capability in generalizing more complex cases to simpler, healthier ones. For example, it attains 85.3% accuracy in the axial plane with training classes (1, 2, 4) and testing classes (0, 3), demonstrating its ability to handle less common scenarios. In the sagittal plane, it achieves 82.1% accuracy with training classes (1, 2, 3) and testing classes (0, 4), leveraging the absence of abnormalities for better outcomes.

However, the model struggles in the coronal view, with only 71.0% accuracy, indicating a need for improvement. The integration of the Prototypical Network, ResNet-50, and federated learning increases computational demands, posing challenges for training and deployment in resource-limited settings. Performance also varies with training and testing class combinations, which represent different knee injury conditions (from no abnormalities to ACL and meniscus tears). For instance, axial view accuracy drops to 60.1% when trained on classes (0, 1, 2) and tested on (3, 4), highlighting the need for careful class selection to optimize results.

The similarity between labels 3 and 4 both involving ACL tears but differing by meniscus tears generates challenges for the proposed model. Their overlapping features cause ambiguity during training and testing, reducing accuracy and precision. When trained on both labels, the model struggles to differentiate ACL-only injuries from those with meniscus damage, increasing misclassification risk. For example, in the axial view, accuracy drops to 60.1% when trained on labels (0, 1, 2) and tested on (3, 4), highlighting the difficulty in distinguishing similar injury types. In a centralized learning setup, different backbone architectures were compared. ResNet-50 outperformed others, achieving 85.3% accuracy in the axial view compared to ResNet-10 (74.1%), ResNet-18 (80.4%), and ResNet-34 (76.7%). Similar leanings held in sagittal and coronal views, confirms ResNet-50’s superiority for diagnostic performance. The proposed method outperforms in diagnostic accuracy, generalization across MRI planes, and patient privacy via federated learning. However, it faces limitations, including lower coronal view performance and high computational demands due to its complex architecture.

## 6 Limitations and future work

### 6.1 Dataset dependence and bias

The model was trained and evaluated on the MRNet dataset, which is collected from a single clinical source. This limits the model’s generalizability to diverse populations, imaging equipment, and clinical protocols. The homogeneity of the dataset also makes the model’s ability to handle real-world inconsistency in MRI quality and acquisition settings.

### 6.2 Generalizability and validation

Future work should incorporate cross-validation strategies and include different datasets from multiple institutions. This would also help assess the model’s ability for domain adaptation and reduce the risk of overfitting to a single source.

### 6.3 Overfitting and domain shift

Although the model shows better performance on MRNet validation data, the risk of overfitting is possible due to the limited variability in imaging. Techniques such as domain generalization, adversarial learning, or contrastive regularization can be explored to address this issue.

### 6.4 Comparison with broader ligament injury detection

While the proposed model analyzed ACL injuries, its applicability to other ligament injuries such as PCL or MCL remains untested. According to Biz et al. (2022) different ligaments exhibit distinct imaging signatures and biomechanical stress profiles, this may require personalized feature extraction strategies. Extending the proposed method to multi-ligament classification and evaluating performance differences is an important task for future research.

### 6.5 Data heterogeneity and real-world utility

MRI data can vary considerably across institutions in terms of resolution, orientation, and scanner hardware. These heterogeneities pose challenges for deployment and require robust domain adaptation techniques. Additionally, clinician-in-the-loop testing and usability assessments will be necessary to validate the model’s practical utility.

## 7 Conclusion

The proposed work investigated the use of our hybrid federated few-shot system for multi-label classification of knee injuries, such as ACL tear and meniscus tear, using MRI images. Prototypical Network was employed with a pre trained 3DResNet50 backbone on the MRNet dataset. This study proposed a novel hybrid approach integrating few-shot learning and federated learning for knee injury diagnosis from MRI scans. Utilizing a 3D ResNet-50 backbone, the model enhances feature extraction and embedding quality. The Centralized and Federated Few-Shot System adopts an episodic-intermittent learning strategy with a Prototypical Network, employing a structured training protocol with SGD optimization, Cross Entropy Loss, and a MultiStep LR scheduler to ensure effective few-shot classification. The proposed approach achieved an accuracy of 85.3% in the axial plane, 82.1% in the sagittal plane, and 70.1% in the coronal plane in the centralized few-shot system. In the federated few- shot system, it achieved 83% in the axial plane, 83.9% in the sagittal plane, and 65.1% in the coronal plane.

## Data Availability

Publicly available datasets were analyzed in this study. This data can be found here: https://stanfordmlgroup.github.io/competitions/mrnet/.
